# Supplementary material for: Optimal Population-Level Infection Detection Strategies for Malaria Control and Elimination in a Spatial Model of Malaria Transmission
Source: PLoS Comput Biol. 2016 Jan 14;12(1):e1004707. doi: 10.1371/journal.pcbi.1004707 (PMC4713231; doi:10.1371/journal.pcbi.1004707)
Supplement: S1 Fig — (PDF) [file pcbi.1004707.s001.pdf]

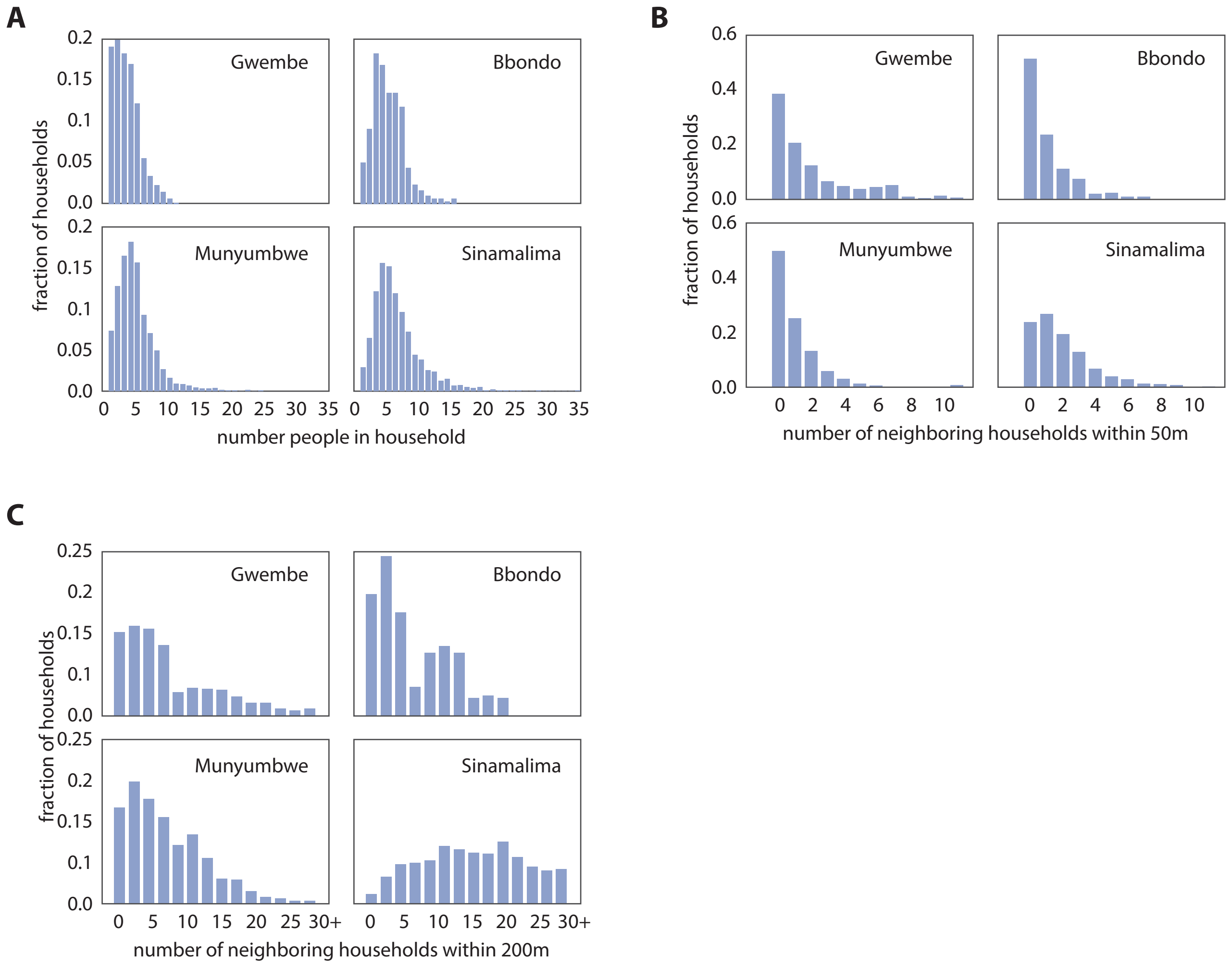

Figure S1. Demographic and geographic features of study area households. (A) Distribution of number of people per household by HFCA. (B) Distribution of number of neighboring households within 50m of each household by HFCA. (C) Distribution of number of neighboring households within 200m of each household by HFCA.
