# Supplementary material for: Optimal Population-Level Infection Detection Strategies for Malaria Control and Elimination in a Spatial Model of Malaria Transmission
Source: PLoS Comput Biol. 2016 Jan 14;12(1):e1004707. doi: 10.1371/journal.pcbi.1004707 (PMC4713231; doi:10.1371/journal.pcbi.1004707)
Supplement: S2 Fig — (PDF) [file pcbi.1004707.s002.pdf]

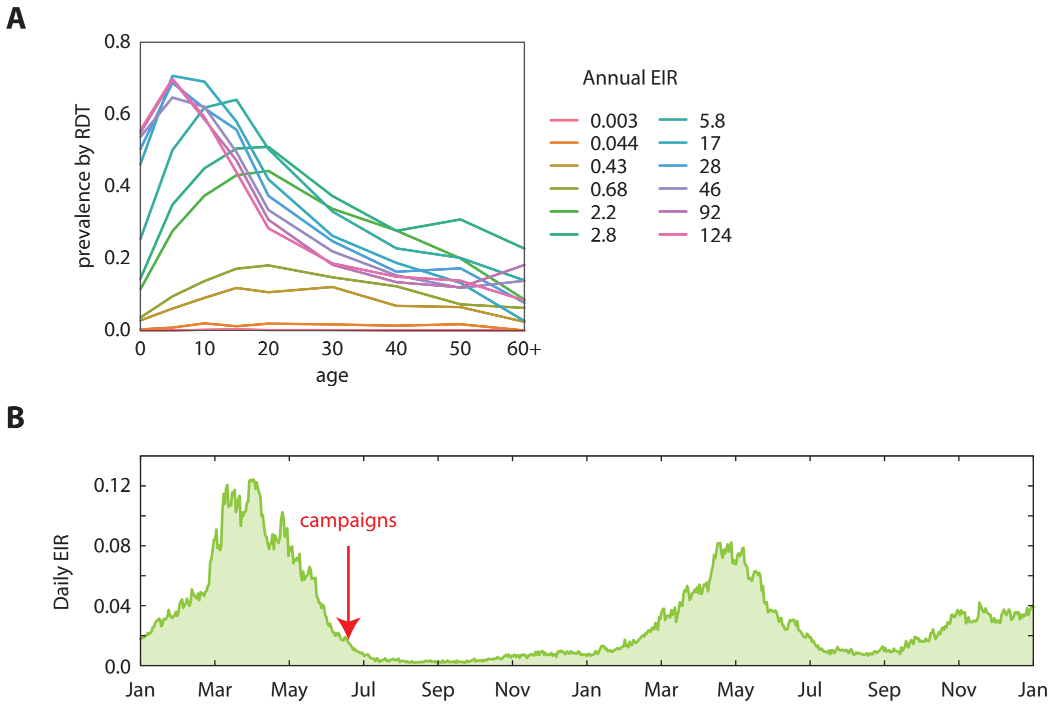

Figure S2. Simulation RDT prevalence by age and seasonal malaria transmission. (A) RDT prevalence by age on June 15 for twelve simulated transmission intensities. (B) Seasonal malaria transmission modeled under southern Zambia climate. Drug campaigns take place on June 15, at the beginning of the dry season.
