# Supplementary figures and images for: Optimal Population-Level Infection Detection Strategies for Malaria Control and Elimination in a Spatial Model of Malaria Transmission
Source: PLoS Comput Biol. 2016 Jan 14;12(1):e1004707. doi: 10.1371/journal.pcbi.1004707 (PMC4713231; doi:10.1371/journal.pcbi.1004707)

**A**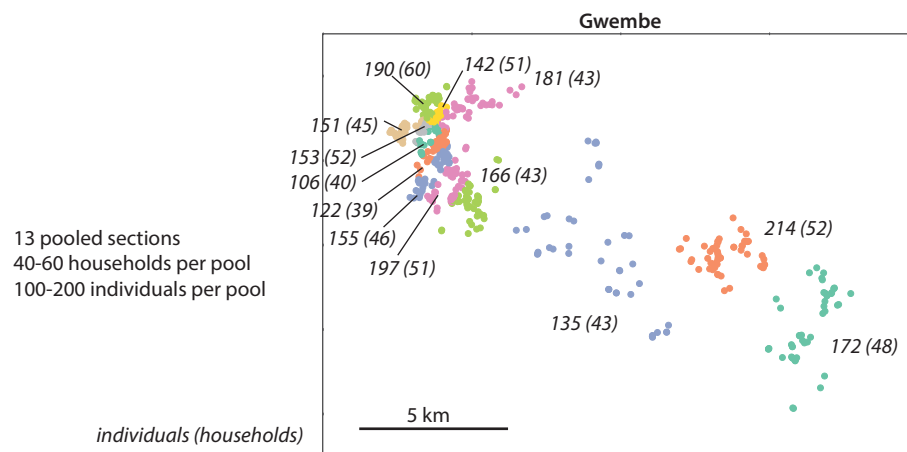**B**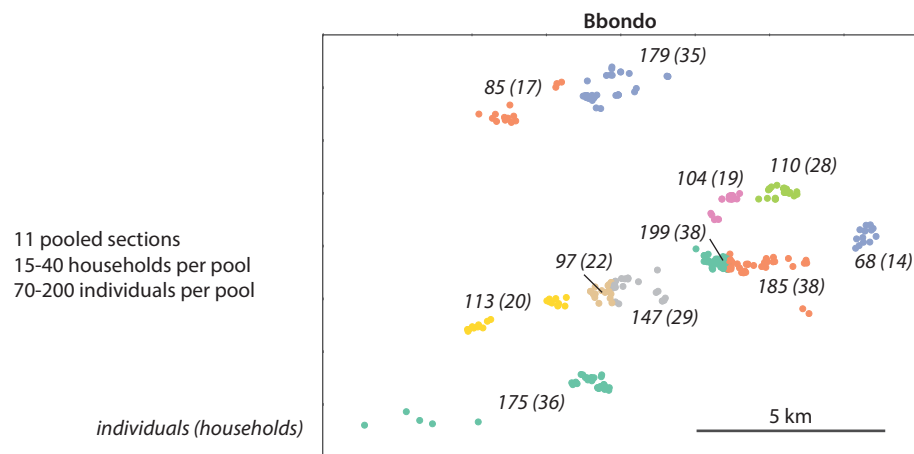

Figure S3. Neighborhood pools used for pooled PCR testing in (A) Gwembe HFCA and (B) Bbondo HFCA.

Supplement: S3 Fig — (PDF) [file pcbi.1004707.s003.pdf]
