# Supplementary material for: Optimal Population-Level Infection Detection Strategies for Malaria Control and Elimination in a Spatial Model of Malaria Transmission
Source: PLoS Comput Biol. 2016 Jan 14;12(1):e1004707. doi: 10.1371/journal.pcbi.1004707 (PMC4713231; doi:10.1371/journal.pcbi.1004707)
Supplement: S4 Fig — (PDF) [file pcbi.1004707.s004.pdf]

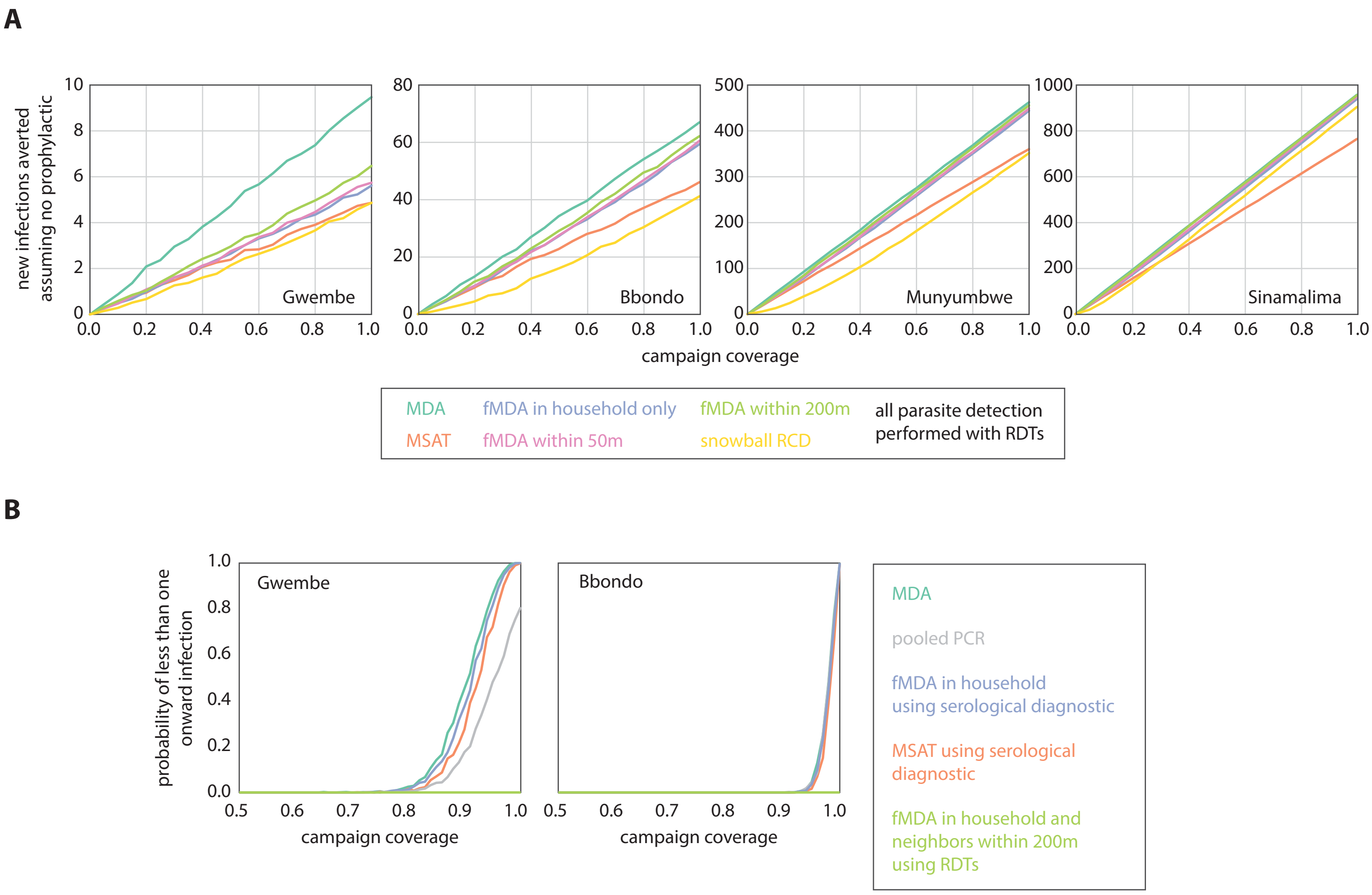


Figure S4. Campaigns without a long-lasting prophylactic are much less successful at averting new infections. (A) Success of infection detection strategies at averting new infections when campaign drug is not prophylactic. Mean of 100 stochastic realizations per coverage level. HFCA populations normalized to 1000. (B) Probability of fewer than 1 onward infection per 1000 people in Gwembe and Bbondo HFCAs if the campaign drug has no prophylactic effect. Mean of 1000 stochastic realizations.
