# Supplementary material for: Optimal Population-Level Infection Detection Strategies for Malaria Control and Elimination in a Spatial Model of Malaria Transmission
Source: PLoS Comput Biol. 2016 Jan 14;12(1):e1004707. doi: 10.1371/journal.pcbi.1004707 (PMC4713231; doi:10.1371/journal.pcbi.1004707)
Supplement: S5 Fig — (PDF) [file pcbi.1004707.s005.pdf]

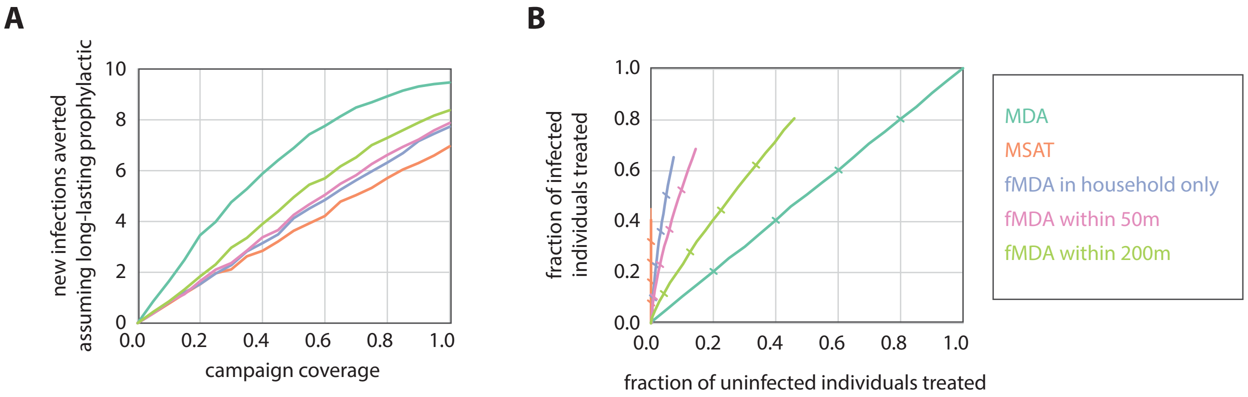

Figure S5. Outcomes of RDT-dependent campaigns in Gwembe HFCA using an improved RDT with sensitivity of 10 parasites/µL. (A) Success of infection detection strategies at averting new infections. Mean of 100 stochastic realizations per coverage level. HFCA populations normalized to 1000. (B) Success of infection detection strategies at finding infected individuals while minimizing overtreatment. Mean of 100 stochastic realizations per coverage level. Ticks indicate every 20% of coverage.
