# Supplementary material for: Optimal Population-Level Infection Detection Strategies for Malaria Control and Elimination in a Spatial Model of Malaria Transmission
Source: PLoS Comput Biol. 2016 Jan 14;12(1):e1004707. doi: 10.1371/journal.pcbi.1004707 (PMC4713231; doi:10.1371/journal.pcbi.1004707)
Supplement: S6 Fig — (PDF) [file pcbi.1004707.s006.pdf]

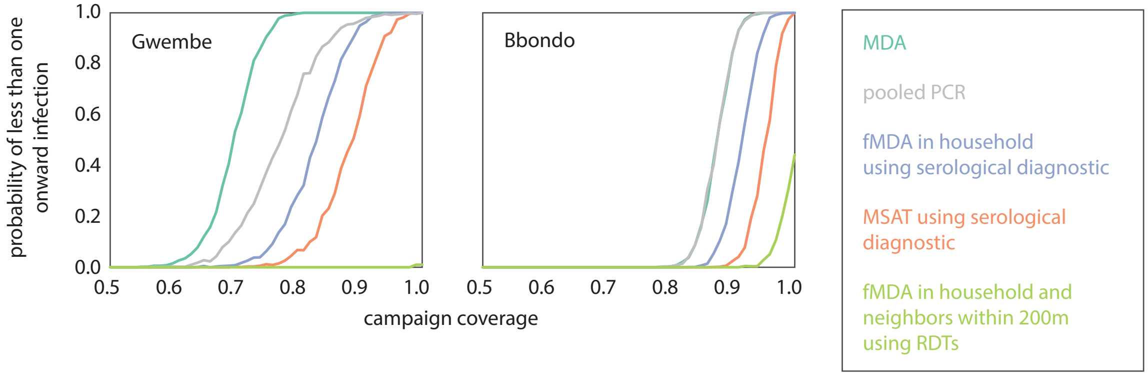

Figure S6. Lkelihood of interrupting transmission increases if vectors are not assumed to remain close to the source of their infection. Probability of fewer than 1 onward infection per 1000 people in Gwembe and Bbondo HFCAs if infected vectors are free to bite anyone in the HFCA rather than tending to remain close to the household where they were infected (all weights set to 1 in equation 3). Mean of 1000 stochastic realizations.
