# Supplementary material for: Optimal Population-Level Infection Detection Strategies for Malaria Control and Elimination in a Spatial Model of Malaria Transmission
Source: PLoS Comput Biol. 2016 Jan 14;12(1):e1004707. doi: 10.1371/journal.pcbi.1004707 (PMC4713231; doi:10.1371/journal.pcbi.1004707)
Supplement: S7 Fig — (PDF) [file pcbi.1004707.s007.pdf]

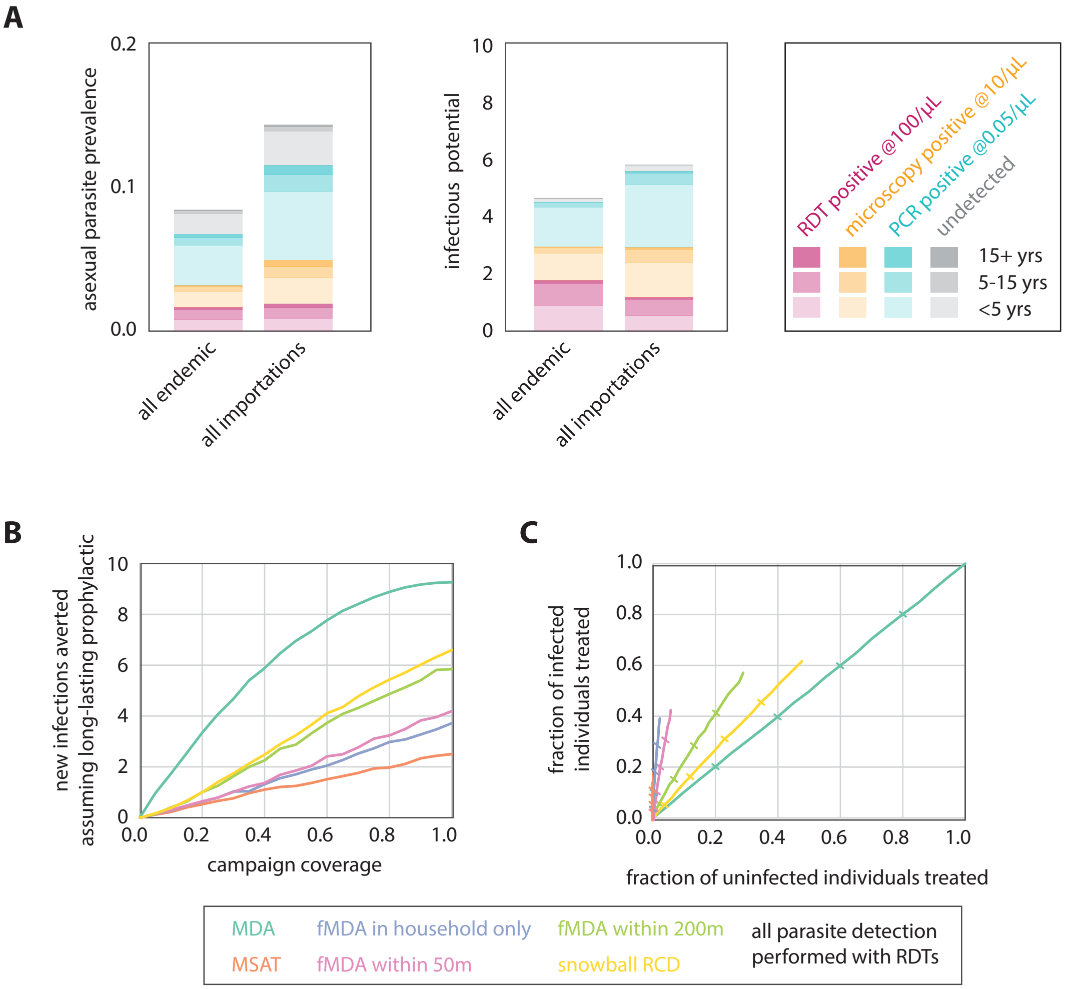

Figure S7. Infection detection in Gwembe HFCA if all infections are importations. For the imported case, all members of households with at least one RDT+ individual are modeled as recent immigrants from a region of EIR = 50. (A) True asexual parasite prevalence and infectious potential if all RDT positive infections are endemic or imported. Infectious potential defined as in Figure 2C. Mean of 100 stochastic realizations. (B) Success of infection detection strategies at averting new infections. Mean of 100 stochastic realizations per coverage level. HFCA population normalized to 1000. (C) Success of infection detection strategies at finding infected individuals while minimizing overtreatment. Mean of 100 stochastic realizations per coverage level. Ticks indicate every 20% of coverage.
